# *Faecalibacterium duncaniae* as a novel next generation probiotic against influenza

**DOI:** 10.3389/fimmu.2024.1347676

**Published:** 2024-03-12

**Authors:** Loïc Chollet, Séverine Heumel, Lucie Deruyter, Fabrice Bouilloux, Lou Delval, Véronique Robert, Marie-Hélène Gevaert, Muriel Pichavant, Valentin Sencio, Cyril Robil, Isabelle Wolowczuk, Harry Sokol, Sandrine Auger, Alexandre Douablin, Philippe Langella, Jean-Marc Chatel, Corinne Grangette, François Trottein

**Affiliations:** ^1^ Univ. Lille, Centre National de la Recherche Scientifique (CNRS), Institut National de la Santé et de la Recherche Médicale (Inserm), Centre Hospitalier Universitaire (CHU) Lille, Institut Pasteur de Lille, U1019-Unité Mixte de Recherche (UMR) 9017 - CIIL – Centre d′Infection et d′Immunité de Lille, Lille, France; ^2^ Biomnigene Société Anonyme (SA), Besançon, France; ^3^ Unité Mixte de Recherche 1319 (UMR1319) Micalis, Université Paris-Saclay, Institut National de Recherche Pour l'Agriculture, l'Alimentation et l'Environnement (INRAE), AgroParisTech, Jouy-en-Josas, France; ^4^ Centre National de la Recherche Scientifique (CNRS), Institut National de la Santé et de la Recherche Médicale (Inserm), Centre Hospitalier Universitaire (CHU) Lille, Univ. Lille, Institut Pasteur de Lille, US 41-UAR 2014-PLBS, Lille, France; ^5^ Sorbonne Université, Institut National de la Santé et de la Recherche Médicale (INSERM), Centre de Recherche Saint-Antoine, Centre de Recherche scientifique Saint-Antoine (CRSA), Assistance Public – Hôpitaux de Paris (AP-HP), Saint-Antoine Hospital, Gastroenterology Department, Paris, France; ^6^ Paris Center for Microbiome Medicine (PaCeMM) Fédérations Hospitalo-Universitaires (FHU), Paris, France

**Keywords:** influenza, live biotherapeutic products, Faecalibacterium, interferons, microbiota, SCFAs

## Abstract

The gut-lung axis is critical during viral respiratory infections such as influenza. Gut dysbiosis during infection translates into a massive drop of microbially produced short-chain fatty acids (SCFAs). Among them, butyrate is important during influenza suggesting that microbiome-based therapeutics targeting butyrate might hold promises. The butyrate-producing bacterium *Faecalibacterium duncaniae* (formerly referred to as *F. prausnitzii*) is an emerging probiotic with several health-promoting characteristics. To investigate the potential effects of *F. duncaniae* on influenza outcomes, mice were gavaged with live *F. duncaniae* (A2-165 or I-4574 strains) five days before infection. Supplementation of *F. duncaniae* was associated with less severe disease, a lower pulmonary viral load, and lower levels of lung inflammation. *F. duncaniae* supplementation impacted on gut dysbiosis induced by infection, as assessed by 16S rRNA sequencing. Interestingly, *F. duncaniae* administration was associated with a recovery in levels of SCFAs (including butyrate) in infected animals. The live form of *F. duncaniae was* more potent that the pasteurized form in improving influenza outcomes. Lastly, *F. duncaniae* partially protected against secondary (systemic) bacterial infection. We conclude that *F. duncaniae* might serve as a novel next generation probiotic against acute viral respiratory diseases.

## Introduction

Influenza is a seasonal, acute, communicable, epidemic respiratory illness caused by influenza viruses (1–3). It has been estimated that influenza leads to up to 500,000 deaths per year worldwide (4). Every 20 to 30 years, novel influenza strains (mostly influenza A viruses, IAVs) emerge in humans (after transmission from an animal host) and cause pandemic disease (5). Influenza viruses affect the upper and lower respiratory tracts; the resulting illness is sometimes severe, as characterized by intense fever, cough, headache, and lung inflammation. In some cases, influenza is followed by secondary bacterial infection or other severe complications (6). Vaccination is still the best way of preventing influenza infections and/or to reducing the severity of the disease. However, the effectiveness of vaccines against seasonal influenza varies significantly, and only 40% to 60% of the vaccinated general population are protected (7). As a result of immunosenescence and immune system suppression or deficiency, older adults and immunocompromised individuals are less well protected and thus more likely to experience a poor disease outcome (including death, in some cases) (8). Hence, new approaches for preventing and managing on influenza epidemics and pandemics are highly desirable.

The gut harbors trillions of microbes; this complex microbial ecosystem has a crucial role in human health via a variety of physiological mechanisms (e.g. strengthening of the gut barrier, stimulation of the host’s metabolism, and immune regulation) (9). A growing body of evidence indicates that the gut microbiota profoundly influences the host’s immunity against respiratory tract viruses and the latter’s pathogenicity; conversely, viral respiratory diseases adversely impact the gut microbiota’s composition and function (10–14). One can reasonably hypothesize that manipulation of the gut-lung axis can influence the management of influenza infections. The results of preclinical studies suggest that antibiotic treatment and thus functional dysbiosis of the gut microbiota enhance susceptibility to influenza (15). Conversely, shaping the intestinal microbiota with probiotics or prebiotics might be instrumental in protecting the body against severe influenza (16–19). In mechanistic terms, the gut microbiota influences the lungs’ defenses and controls pulmonary disease outcomes through a broad panel of microbial metabolites and membrane components (20). The short chain fatty acids (SCFAs) produced by bacterial fermentation are likely to be important factors in this process (12, 21, 22). The main SCFAs produced (acetate (C2), propionate (C3), and butyrate (C4)) have a major impact on health - notably by influencing the host immune responses in and beyond the gastrointestinal tract (including in the lung) (23, 24). It was recently shown that acetate controls the IAV load in the lung and that this effect depended on the free fatty acid receptor 2, the NLRP3 inflammasome, and type I interferons (IFNs) (25). This finding corroborated earlier reports on the protective role of acetate during a respiratory syncytial virus infection (26, 27). It is noteworthy that acetate also protects against post-influenza secondary bacterial infections (28, 29). In contrast, acetate failed to protect against SARS-CoV-2 infection (30, 31). To the best of our knowledge, the putative role of propionate during influenza has not been documented. Trompette et al. highlighted the importance of butyrate in this context (24). Indeed, animals fed a high-fiber diet or supplemented with butyrate are less susceptible to influenza, and this effect was associated with an enhanced effector CD8^+^ T cell response and less intense lung inflammation. A clinical study of patients undergoing allogeneic hematopoietic stem cell transplantation recently confirmed the important role of butyrate in viral respiratory diseases. In fact, patients with a greater abundance of butyrate-producing bacteria in the gut and elevated fecal butyrate levels were five times less likely to develop viral respiratory diseases (32). The results of clinical studies indicate that infections with respiratory viruses (including IAV and SARS-CoV-2) are associated with a drop in the number of SCFA producers; this may lead to potentially harmful consequences, such as a decrease in numbers of anaerobic butyrate-producing bacteria such as *Faecalibacterium*, a member of the Clostridium IV group of the Bacillota (previously known as Firmicutes) (33–35).

*Faecalibacterium prausnitzii* is a well-known butyrate-producing bacterium. It is one of the most abundant commensals in the healthy human gut and accounts for around 5% of the total fecal microbiota (36–38). The results of several clinical studies have shown that the frequency of *F. prausnitzii* falls dramatically during gut diseases (39–42). We and others have shown that *F. prausnitzii* has a crucial role in gut health and exhibits strong anti-inflammatory effects *in vitro* and *in vivo (* 43–47). A recent study showed that *F. prausnitzii* reduces colorectal colitis in mice by regulating the T regulatory/T helper17 balance (46). More recently, our data in a humanized model showed that *F. prausnitzii* alleviated intestinal inflammation by stimulating IL-10-secreting, Foxp3-expressing T regulatory cells (characterized by simultaneous CD4 and CD8α expression) (48). Mechanistically, *F. prausnitzii*’s anti-inflammatory property has been attributed to the production of metabolites such as butyrate (39, 43, 46, 49, 50) and the microbial anti-inflammatory molecule (MAM) protein (47, 51, 52). Recently, new species of the *Faecalibacterium* genus have been described and some strains including the reference strain A2-165 have been reclassified as *F.duncaniae (* 53). With a view to determining the role of gut butyrate in respiratory viral infections, we investigated the potential protective effect of *F. duncaniae* strains [notably the reference strain A2-165 and the strain I-4574, shown to produce equal amount of butyrate (54)] in an experimental model of influenza. Our results demonstrate that the prophylactic treatment of mice with *F. duncaniae* provides protection against influenza by limiting body weight loss, pulmonary viral load, lung/gut injury and secondary (systemic) bacterial infection. These protective effects were associated with a return to pre-influenza levels of SCFAs. The live form of *F. duncaniae* was more potent than the pasteurized form in alleviating influenza outcomes. Our findings might have applications in the clinical management of viral respiratory diseases.

## Results

### Oral administration of *F. duncaniae* protects against experimental influenza

**Figure 1:**
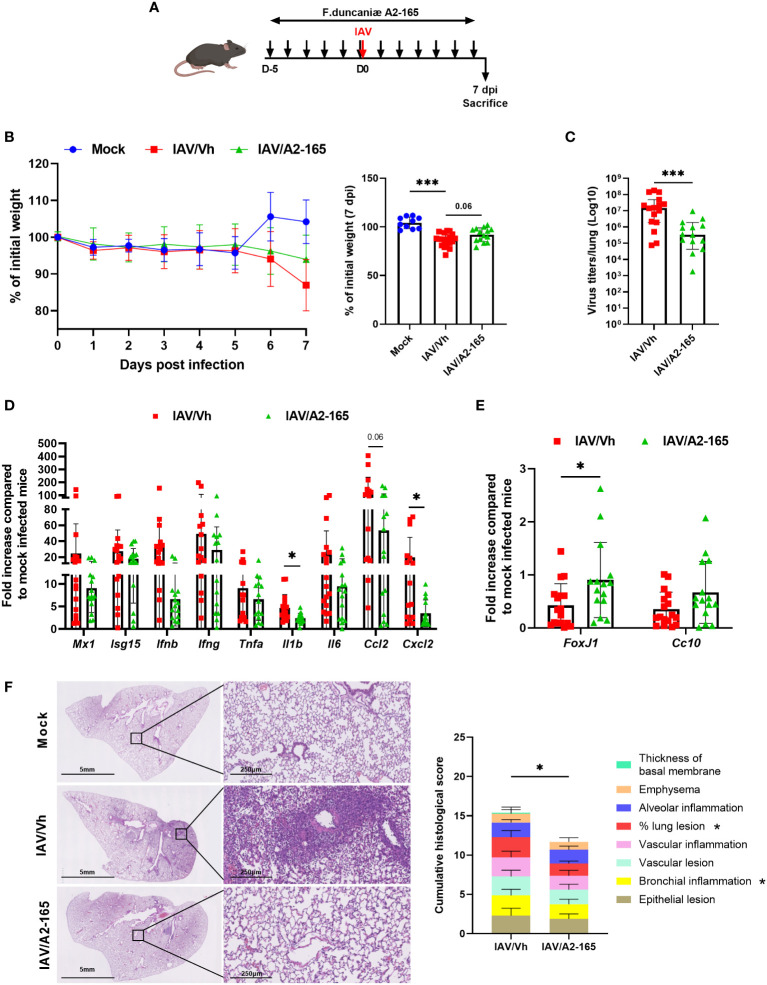
Effect of *F*. *duncaniae* A2-65 administration prior to influenza infection on body weight loss, and lung viral load and inflammation. **(A)** Schematic representation of the experimental procedure. Male C57BL/6 mice received PBS-glycerol (Vh) or A2-65 (5 x 10^9^ CFU/mouse/day) in PBS-glycerol, 5 days before IAV infection and until the sacrifice. Mice were infected with IAV on day 0 and were sacrificed on 7 dpi. Non-infected vehicle-treated mice (Mock) served as controls. **(B)** Body weight loss during infection (expressed as percentage initial weight). The right panel shows differences at 7 dpi. **(C)** Impact of *F*. *duncaniae* A2-65 treatment on the pulmonary viral load. The number of infectious particles was quantified using a TCID_50_ assay. Data are expressed as the mean ± SEM of infectious virus particles per lung. The solid lines correspond to the median values. **(D, E)** mRNA copy numbers were quantified by RT-PCR (7 dpi). The data are expressed as the mean fold change relative to average gene expression in mock-infected animals ± SEM. **(F)** *Left* panel, Representative photomicrographs of lungs from IAV-infected mice (hematoxylin and eosin staining) at 7 dpi. **(F)** *Right* panel, Lung pathological scoring. The sum of the subscores is shown (n=8). Statistical analyses were performed on the cumulative score and on individual score parameters. **(B–E)** A pool of two representative independent experiments is shown (n = 15-20). **(F)** a single experiment was performed (n = 8). Significant differences were determined using the two-tailed Mann Whitney *U* test **(C–E)** *left* panel), and One-way ANOVA Kruskal-Wallis test (nonparametric), followed by the Dunn’s posttest test (**(B)** *right* panel) (* *P* ≤ 0.05, ** P < 0.01, *** *P* ≤ 0.001).

We first investigated the effect of the reference *F. duncaniae* strain A2-165, which exhibits strong anti-inflammatory activities (43, 55). C57BL/6 mice were supplemented daily via the intragastric administration of A2-165 or vehicle for five days before the administration of a sub-lethal dose of IAV (**Figure 1A**). The treatment was maintained until sacrifice on day 7 post-infection (7 dpi), the peak of the acute phase. Mock-infected mice served as controls. On average, the IAV-infected mice had lost 12% of their initial body weight on 7 dpi (**Figure 1B**). Supplementation with A2-165 attenuated the body weight loss resulting from infection, although the difference was not statistically significant. We next sought to determine whether A2-165 influenced viral replication in lungs. Determination of the lung infectious titers in a tissue culture infectious dose (TCID_50_) assay indicated that A2-165 treatment was associated with a significantly lower viral load at 7 dpi (**Figure 1C**).

The pathogenesis of viral infection is associated with an excessive inflammatory response. We therefore sought to determine the consequences of *F. duncaniae* A2-165 treatment on lung inflammation, as judged from gene expression assays and a pathology assessment. We first measured the mRNA expression of IFN-stimulated genes (ISGs) and inflammatory genes in the lungs, using quantitative RT-PCR assays. Although it did not reach significance, the level of gene expression of the inducible protein Mx1 - the expression of which is proportional to the virus load at this time – tended to be lower in A2-165-treated animals than in nontreated controls (**Figure 1D**). Accordingly, the mRNA expression levels of type I IFN (IFN-β) and type II IFN (IFN-γ) - both of which were associated with uncontrolled pro-inflammatory processes and the impairment of lung epithelial regeneration at 7 dpi (56) - were lower in A2-165-treated animals. This was also the case for inflammatory genes, such as *Tnfa, Il1b, Il6, Ccl2* and *Cxcl2* (**Figure 1D**). Influenza results in an impairment of the epithelial barrier function (57). Accordingly, the expression of transcripts encoding the transcription factor forkhead box protein J1 (Foxj1, involved in cilium formation) and the Clara cell secretory protein (Cc10, involved in the repair and maintenance of airway integrity) was lower in IAV-infected animals (**Figure 1E**). Interestingly, A2-165 administration significantly attenuated the relative fall in *Foxj1* and *Cc10* (not significant) gene expression - suggesting that administration of the bacterium was associated with reinforcement of the lung barrier. Examination of lung sections from IAV-infected mice revealed significant pathological lesions at 7 dpi (**Figure 1F**, *left* panel). These changes were alleviated in *F. duncaniae*-treated animals, as evidenced by less alveolar wall thickening and less inflammatory cell infiltration of the alveoli. The lung histopathology scores were significantly lower in mice treated with A2-165 (**Figure 1F**, *right* panel). Taken as a whole, these results show that *F. duncaniae* A2-165 not only hampered viral replication in the lungs but also diminished lung inflammation.

### The protective effect of *F. duncaniae* is not strain-specific and associates with enhanced type III IFN-Λ production

**Figure 2:**
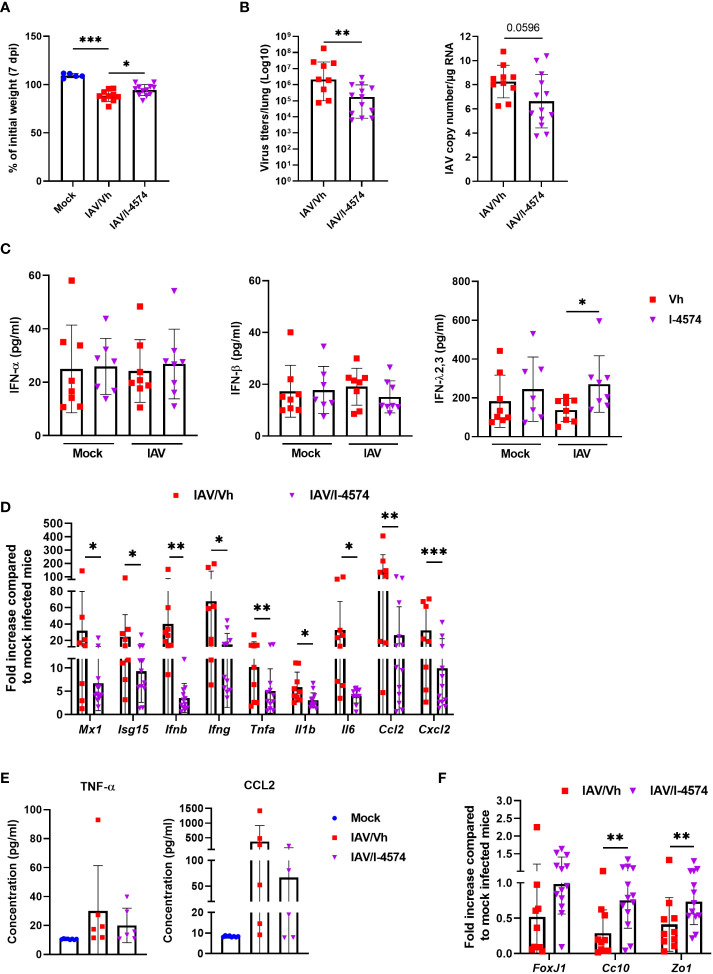
Effect of *F*. *duncaniae* I-4574 administration prior to influenza infection on body weight loss, lung viral load and inflammation, and IFN production in bronchoalveolar lavage fluids. **(A)** Body weight loss at 7 dpi (n = 5, Mock and n = 10-12, IAV)). **(B)** *left* panel, the number of infectious particles was determined using a TCID_50_ assay. Data are expressed as the mean number of infectious virus particles per lung. The solid lines correspond to the median values. *Right* panel, quantification of viral M1 protein transcript level in the lung (qRT-PCR assay). Data are expressed as genome copy/μg RNA (n = 10-13). **(C)** Bronchoalveolar lavage fluids were collected on 2 dpi. Concentrations of IFN-α, IFN-β, and IFN-Λ2,3 are shown (n = 7-8). **(B, F)** mRNA copy numbers were quantified by RT-PCR (7 dpi). Data are expressed as the mean fold change relative to average gene expression in mock-infected animals (n = 10-13). **(E)** BALs were collected on 7 dpi and TNF-α and CCL-2 were quantified by ELISA (n = 7-8). **(B–F)** One of two representative experiments is shown. For all graphs, errors indicate mean ± SEM. Significant differences were determined using the two-tailed Mann Whitney *U* test **(B, D, F)** and One-way ANOVA Kruskal-Wallis test (nonparametric), followed by the Dunn’s posttest test **(A, C, E)** (* *P* < 0.05, ** *P* < 0.01, *** *P* < 0.001).

In order to confirm that *F. duncanie*’s protective effect was not strain-specific, we evaluated the impact of a second strain (I-4574). Supplementation with I-4574 was associated with significantly less body weight loss during influenza, which indicated a better disease outcome (**Figure 2A**). Accordingly, the viral load (as determined by the TCID50) was lower in mice that received I-4574 (**Figure 2B**, *left* panel). Quantification of the M1 protein transcripts in RT-qPCR assays confirmed this finding (**Figure 2B**, *right* panel). To better explore the mechanisms involved in reduced viral load, we evaluated the impact of I-4574 supplementation on the antiviral immune response at early time point (2 dpi). To this end, we measured levels of various IFNs in bronchoalveolar lavage fluids; IFNs are known to be involved in the control of viral replication. Mock-infected mice treated with vehicle or I-4574 were used as controls. While I-4574 had no effect on the production of the type I (IFN-α and IFN-β) and type II (IFN-γ) IFN, it augmented that of the type III IFN IFN-Λ2,3 (**Figure 2C** and not shown). At this early time point, we failed to detect IFN production in mice infected with influenza (without treatment). It is noteworthy that in the absence of IAV, I-4574 also enhanced IFN-Λ2,3 production, albeit not in a significant manner. Quantification of inflammatory gene expression in the lungs at 7 dpi confirmed the efficacy of I-4574 in this model of infection (7 dpi, **Figure 2D**). Accordingly, the BALF concentrations of TNF-α and CCL-2 proteins were lower (albeit not significantly) in I-4574-treated mice than in control mice (**Figure 2E**). Lastly, I-4574 attenuated the lower mRNA expression of markers involved in lung barrier functions, such as *Foxj*, *Cc10*, and the tight junction protein Zonula occludens 1 (*Zo1*) (**Figure 2F**). We conclude that the protective effect of *F. duncaniae* is not strain-specific and is associated with enhanced IFN-Λ production soon after infection.

### *F. duncaniae* treatment induces a distinct shift in gut microbial composition and restores the production of SCFAs during influenza

**Figure 3:**
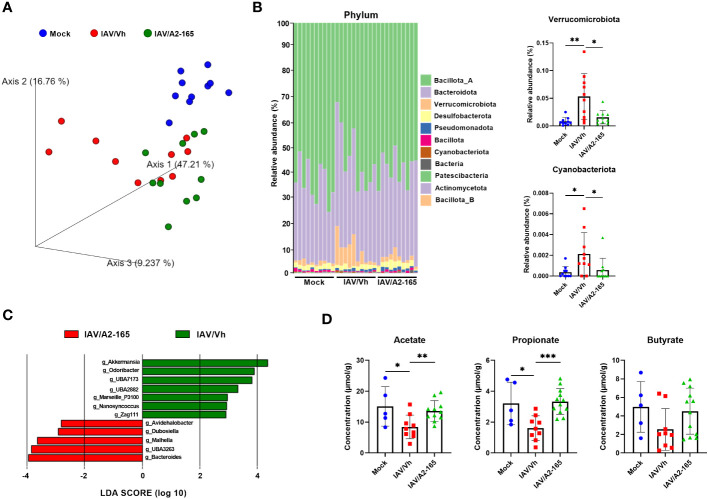
Impact of *F*. *duncaniae* supplementation in IAV-infected mice on the production of SCFAs. **(A)** Seven days after IAV infection, fecal contents were collected for 16S rRNA profiling. Fecal samples from each mock-infected mouse were also collected. Bacterial communities were clustered using PCA of Weighted UniFrac distance matrices (beta diversity). The first three principal coordinates (PC1, PC2 and PC3) are plotted for each sample and the percentage variation in the plotted principal coordinates is indicated on the axes. Each spot represents one sample and each group of mice is denoted by a different color (blue: Mock-infected, red: IAV/Vh, green: IAV/A2-165). Distance between dots represents extent of compositional difference. **(B)** *Left* panel, Taxonomic (phylum) composition of the cecal microbiota. *Right* panel, the relative frequencies of Verrucomicrobiota and Cyanobacteriota are depicted. **(C)** LEfSe analysis indicating the most discriminant bacterial genus associated with changes in *F duncaniae*-treated mice. Only species with a statistically significant LDA score (log10) >2 (compared to vehicle) are shown. **(A–C)** (n = 10/group, one of two independent experiments shown). **(D)** Cecal contents were collected for SCFA quantification. Results are expressed as mean ± SEM (n = 5, mock and n = 9-11, IAV). **(B)** *Right* panel and **(D)** Significant differences were determined using the One-way ANOVA Kruskal-Wallis test (nonparametric), followed by the Dunn’s posttest test (* *P* < 0.05, ** *P* < 0.01, *** *P* < 0.001).

Influenza infection in mice associates with gut dysbiosis at 7 dpi (21, 28, 58–60). To investigate the potential effects of *F. duncaniae* supplementation on gut microbiota’s composition during infection, 16S rRNA gene amplicon sequencing was performed on fecal samples. As expected, IAV induced changes in the composition of the gut microbiota at 7 dpi (**Figure 3**). Weighted Unifrac-based principal component analysis (PCA) plot revealed that the two IAV-infected groups clustered away from the mock-infected group (*P* = 0.004 for the vehicle group and *P* = 0.001 for the *F. duncaniae* group as assessed by permutational multivariate analysis of variance, PERMANOVA) (**Figure 3A** and not shown). The infected group that received *F. duncaniae* separated from the vehicle control group, although not significantly (*P* = 0.136, PERMANOVA). We next analyzed the composition of the gut microbiota at different taxonomic levels. At the phylum level, IAV led to a significant increased relative abundance of Bacteroidota (previously known as Bacteroidetes), Verrucomicrobiota, Pseudomonadota (Proteobacteria) and Cyanobacteriota (**Figure 3B**, *left* panel and **Supplementary Figure 1B**). On the other hand, IAV associated with a drop of Bacillota (Firmicutes). Supplementation with *F. duncaniae* significantly attenuated changes in Verrucomicrobiota and Cyanobacteriota relative proportions (**Figure 3B**). A significant impact of *F. duncaniae* was also observed at lower taxonomic levels (**Supplementary Figure 1C** for the class). Linear discriminant analysis effect size (LEfSe) analyses revealed that, at genus levels, *F. duncaniae* supplementation augmented the relative frequency of the SCFA producing bacteria *Dubosiella* (Bacilllota) and *Muribaculaceae UBA3263* (Bacteroidota) (**Figures 3C, D**). On the other hand, it significantly reduced the IAV-induced augmentation of *Akkermansia* (Verrucomicrobiota), *Odorobacter* (*Bacteroides*) and *crytpobacteroides* (*Bacteroides*). In line with the fact that it does not colonize in the mouse gut (61, 62), we failed to detect *F. duncaniae* by quantitative PCR. Collectively, *F. duncaniae* administration impacted on gut dysbiosis during influenza.

The production of SCFAs depends strongly on the gut microbiota’s functional activity. As observed previously (28, 29), cecal levels of acetate, propionate and butyrate fell massively upon IAV infection (**Figure 3D**). In contrast, levels of isobutyrate, isovalerate and valerate did not change significantly (**Supplementary Figure 1D**). Interestingly, *F. duncaniae* treatment was associated with attenuation of the drop in acetate, propionate and butyrate (not significant) levels at 7 dpi. Given that acetate and butyrate are important during influenza (24, 25, 28, 29, 32), this partial restoration might contribute to the better disease outcomes associated with *F. duncaniae* administration.

### Live *F. duncaniae* is more potent than pasteurized *F. duncaniae* in mitigating negative influenza outcomes

**Figure 4:**
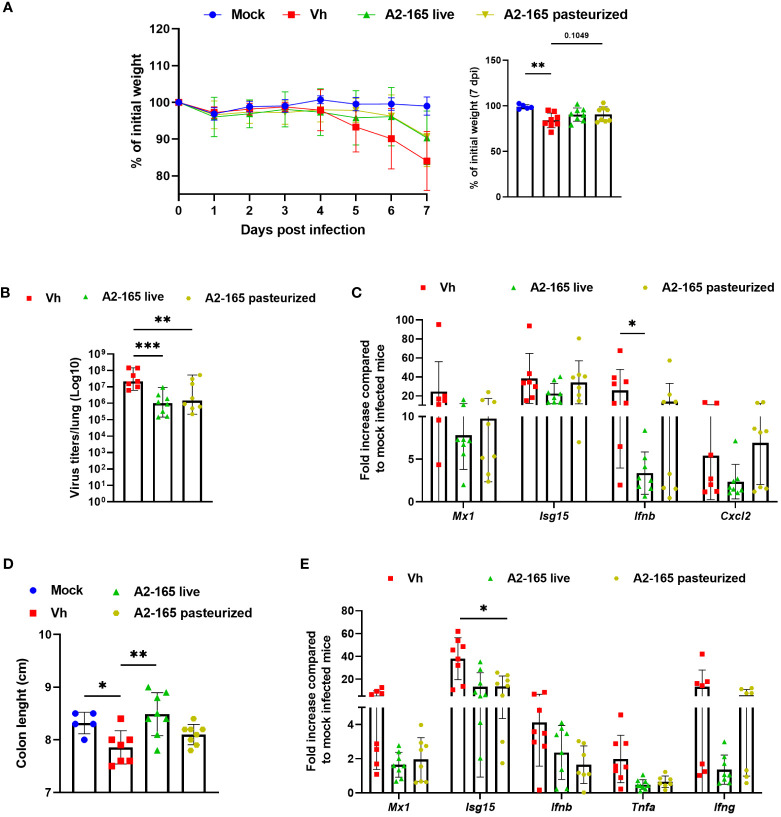
Effect of live and pasteurized *F*. *duncaniae* pre-treatment on body weight loss, lung viral load and inflammation. Mice received PBS-glycerol or A2-65 in PBS-glycerol 5 days before IAV infection and until the sacrifice. **(A)**, Body weight loss during infection (expressed as percentage initial body weight). The right panel shows differences at 7 dpi. **(B)**, the number of infectious particles was determined in a TCID_50_ assay. The data are expressed as the mean number of infectious virus particles per lung. The solid lines correspond to the median values. **(C)**, mRNA copy numbers in lungs were quantified by RT-PCR (7 dpi). The data are expressed as the mean fold change relative to average gene expression in mock-infected animals ± SEM. **(D)**, Colon length measured at 7 dpi. **(E)**, mRNA copy numbers in colons were quantified by RT-PCR (7 dpi). One of two representative experiments is shown (n = 6-8). For all graphs, errors indicate mean ± SEM. Significant differences were determined using One-way ANOVA Kruskal-Wallis test (nonparametric), followed by the Dunn’s posttest test (* *P* < 0.05, ** *P* < 0.01, *** *P* < 0.001).

To investigate how metabolites (*e.g*. butyrate) might contribute to the *F. duncaniae*’s protective influence, we metabolically inactivated bacteria by pasteurization. Treatment with the pasteurized bacteria was associated with an attenuation (not significant) of body weight loss in IAV-infected mice (**Figure 4A**). Although the pasteurized form was less efficacious than the live form, it was associated with a significantly lower viral load in the lungs (**Figure 4B**). Accordingly, the expression level of gene encoding Mx1, Isg15 and IFN-β was lower in mice supplemented with pasteurized *F. duncaniae* than in control (vehicle) mice (**Figure 4C**). Here again, the effects were less strong than with live *F. duncaniae*, particularly for IFN-β. Pasteurized *F. duncaniae* failed to influence the expression of transcripts encoding inflammatory cytokines such as CXCL2 (**Figure 4C** and data not shown). Influenza infection is known to be associated with gut injury, poor mucosal barrier function, and greater levels of gut inflammation (29, 58, 63). Given that *F. duncaniae* has been linked to lower levels of gut inflammation, we compared the effects of live vs. pasteurized *F. duncaniae* in IAV-infected mice. The colon length - a marker of intestinal inflammation - was lower in IAV-infected mice (**Figure 4D**). Interestingly, live *F. duncaniae* (but not pasteurized *F. duncaniae*) abrogated the IAV-induced reduction in colon length. Influenza led to greater expression of ISGs and inflammatory genes in the colon (**Figure 4E**). Live *F. duncaniae* and, to a lesser extent (for *Ifng*) pasteurized *F. duncaniae*, abrogated cytokine production, as assessed in quantitative RT-PCRs (**Figure 4E**). We conclude that the live form of *F. duncaniae* alleviates influenza outcomes more potently than the pasteurized form does.

### *F. duncaniae* treatment is associated with fewer systemic bacterial infections after influenza

**Figure 5:**
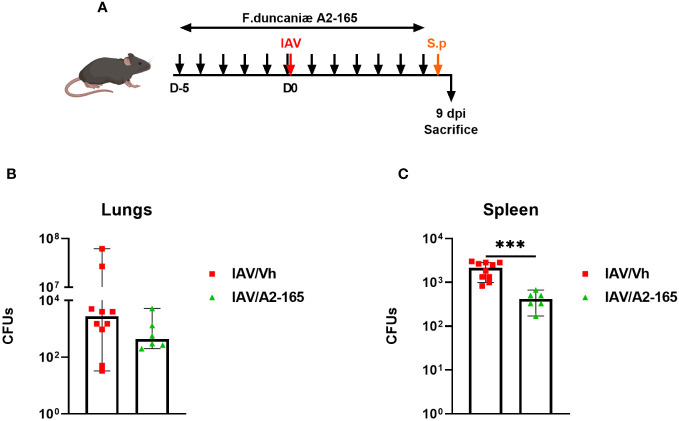
Effect of live *F*. *duncaniae* pre-treatment on bacterial superinfection post-influenza. **(A)** Schematic representation of the experimental procedure. Mice received PBS-glycerol or A2-65 in PBS-glycerol, 5 days before IAV infection and until the sacrifice. Mice were challenged intranasally with *S. pneumoniae* 7 days post IAV infection (1x10^3^ c.f.u). **(B, C)** The number of bacteria in the lungs and the spleen was determined 30 hours after the *S. pneumoniae* challenge. The solid lines correspond to the median values (n = 6-10). For all graphs, errors indicate mean ± SEM. Significant differences were determined using the two tailed Mann Whitney *U* test (***P < 0.001).

Individuals with severe influenza are prone to develop secondary bacterial infections. This enhanced susceptibility is due - at least in part - to impaired antibacterial defenses and altered lung integrity and barrier functions (64, 65). We showed recently that the drop in acetate production during IAV causes bacterial superinfection (28, 29). In view of *F. duncaniae*’s effect on SCFA levels, we next investigated the bacterium’s potential effect on secondary bacterial infections after influenza. To this end, *F. duncaniae*-supplemented, IAV-infected mice were infected secondarily with *Streptococcus pneumoniae* (**Figure 5A**). The number of viable bacteria in the lung was counted 30 hours after the inoculation of *S. pneumoniae*. As shown in **Figure 5B**, supplementation of *F. duncaniae* failed to significantly lower the number of viable bacteria in the lung. In the model studied here, *S. pneumoniae* disseminates in the blood to reach the spleen. Of interest, supplementation of *F. duncaniae* was associated with significantly less systemic bacterial translocation in doubly infected mice (**Figure 5C**). This reduced systemic invasion is superinfected mice probably relies on improved lung integrity. We conclude that preventive supplementation with *F. duncaniae* in mice lowers the severity of both influenza and bacterial superinfection.

## Discussion

The dysregulation of the microbiota-gut-lung axis during influenza, consequent to gastrointestinal disorders and dysbiosis (with reduced SCFA levels), can lead to disease severity and secondary outcomes (12, 21, 28, 58–60), which underscores the need for new interventions. In the present study, we investigated preventive supplementation of the butyrate producer commensal *F. duncaniae* as a possible way of ameliorating influenza outcomes. *F. duncaniae* holds great promise as a next-generation probiotic for controlling pathological situations in humans [*e.g*. gut disorders and diabetes (55, 66)]. Given that *F. duncaniae* strongly impacts the gut’s ecological network (67), we hypothesized that supplementation with this bacterium would restore SCFA levels [which are dramatically impacted by influenza (28, 29)] in diseased animals. We found that orally administered *F. duncaniae* partially protected against IAV infection, as reflected by a lower body weight loss, a lower viral load in the lungs, and lower levels of pulmonary and gut inflammation. It is important to note that supplementation with *F. duncaniae* also alleviated systemic secondary bacterial infections. These protective effects were associated with a change in gut microbiota composition and an augmentation of SCFA levels in diseased animals.

The results of several studies have revealed that the use of probiotics (mostly from the *Lactobacillus* and *Bifidobacterium* genera) is beneficial during influenza (18, 68–72). Some probiotics can prophylactically prime antiviral responses in the lungs. These effects are due to both innate and adaptive immune responses. Interestingly, the probiotic regulatory pathway might be closely linked to type I IFNs (71–75). This is in line with other evidence of the key role of the gut microbiota in type I IFN-mediated antiviral immunity (15, 25, 76–80). In our experimental setting, *F. duncaniae* had a significant impact on the viral load, which was associated with greater production of type III IFNs but not of type I IFNs. The continuous (7-day) administration of *F. duncaniae* was indeed associated with a greater IFN-Λ concentration in BALF and this was significant after IAV infection. It is noteworthy that *Bifidobacterium longum* and *Clostridium butyricum* were shown to inhibit IAV replication through IFN-Λ(81, 82). Mechanistically, gut microbiome-induced ω-3 fatty acid 18-hydroxy eicosapentaenoic acid promoted IFN-Λ production by lung epithelial cells (82). Although the mechanisms in our settings remained to be investigated, the present study is (to the best of our knowledge) the first to have shown that the commensal *F. duncaniae* exerts antiviral effects. SCFAs might have an important role in this setting. Indeed, recent research findings indicate that acetate reduces the viral load via an influence on type I IFN (25–27, 83, 84). The corresponding results for butyrate are subject to debate. A study has shown that butyrate favors the type I IFN production and ISG expression (84), whilst others have indicated a negative effect (85, 86). To the best of our knowledge, the potential impact of SCFAs on type III IFN production has not been determined and is thus worthy of further investigation. Other metabolites (76, 82) and certain bacterial components might also be involved in our setting. The antiviral impact of *F. duncaniae* might extend beyond microbiota metabolism. Whether *F. duncaniae* antigenicity (*e.g*. molecular mimicry) plays a part is still an open question. Treatment with *F. duncaniae* during influenza strongly reduces lung and gut inflammation. Whilst the observed, positive effect of *F. duncaniae* in influenza-associated gut disorders is in line with the results of other studies (43, 46, 47), the remote effect on lung inflammation is novel. It would be interesting to investigate the effect of *F. duncaniae* supplementation on other infectious and non-infectious lung diseases. There are several possible mechanisms by which *F. duncaniae* could remotely lower lung inflammation. In this context, SCFAs - which are well known to attenuate lung diseases (20, 21) - are likely to be critical. The effects might also be independent of SCFAs because pasteurized *F. duncaniae* also mitigated negative influenza outcomes (albeit less efficaciously that live *F. duncaniae*) in our experimental settings. Mechanisms leading to reduced pathology upon *F. duncaniae* treatment remain to be studied. Lastly, upon a *S. pneumoniae* challenge, *F. duncaniae* lowered bacterial dissemination into peripheral organ. Acetate might be critical in this setting, as our previously data suggested (28). The later finding is important because bacterial superinfection is the main cause of death in patients with severe influenza (64). It is noteworthy that preventive supplementation with *L. paracasei* and *Blautia faecis* also reduced bacterial superinfection post-influenza (87, 88).

The potential benefits of manipulating the gut microbiome and thus preventing the outcomes of severe viral respiratory diseases such as influenza and coronavirus disease 2019 (COVID-19) have been well established in preclinical models and (to a lesser extent) in humans [for reviews, see (16, 89)]. In COVID-19, a potential benefit of *F. duncaniae* and butyrate has been suggested (90, 91). Our present results suggest that *F. duncaniae* - a major, canonical, butyrate-producing human commensal - might serve as a novel next generation probiotic against viral respiratory diseases. Our study had a number of limitations. Firstly, we focused on sub-lethal influenza using male C57BL/6 mice. The potential effect of *F. duncaniae* on lethal influenza remains to be determined (as well as its effect on female mice). Secondly, it would be interesting to study the effect of *F. duncaniae* on lung pathology and mouse survival upon a secondary bacterial infection. Thirdly, the effect of a prophylactic treatment, and not a therapeutic treatment, was studied. Lastly, we focused on the severe, acute influenza and not on the disease’s long-term effects. This is a relevant issue because the frequency of *F. duncaniae* in the gut is inversely correlated with long COVID-19 (34). It would be interesting to investigate variables in chronic lung disease in influenza-infected mice (92). Secondly, we did not formally identify the mechanism(s) associated with *F. duncaniae*-mediated protection in our model, although the SCFA-IFN pathway is likely to be involved, at least in antiviral effects. It remains to be seen whether altered gut microbiota’s functionality due to *F. duncaniae* supplementation during influenza is causal in the beneficial effects described in this report. In conclusion, the present study demonstrated the potential value of *F. duncaniae* treatment (as an adjuvant to influenza vaccines and anti-influenza drugs) in the prevention of influenza epidemics.

## Material and methods

### Mice and infection

C57BL/6J male mice (8-week-old) were purchased from Janvier-Labs (Le Genest-Saint-Isle, France). Mice were housed under specific pathogen-free condition in the biosafety level 2 animal facility of the Institut Pasteur de Lille. Animals were maintained with a strict 12-hour dark/light cycle and were given *ad libitum* access to regular chow and water. Mice were fed a standard rodent chow (SAFE A04) (SAFE, Augy, France) and water ad libitium. This diet contains11.8% fiber including 10% water-insoluble fiber (3.6% cellulose) and 1.8% water-soluble fiber. After a one-week acclimation period, mice were treated with *F. duncaniae* (see below) and then infected as follows. Mice were anesthetized by intraperitoneal injection of 1.25 mg of ketamine plus 0.25 mg of xylazine in 200 µl of phosphate buffered saline (PBS), and then intranasally (i.n.) infected with 50 µl of PBS containing (or not, for mock group) 75 plaque forming units (p.f.u.) of the H3N2 IAV strain A/Scotland/20/1974. For the superinfection, mice were intranasally infected with *S. pneumoniae* (serotype 1, clinical isolate E1586, 1x10^3^ viable bacteria) 7 days post-influenza (28). Mice were euthanized by cervical dislocation. The number of live bacteria in lungs and spleen was determined 30 hours after *S. pneumoniae* challenge as described (28). All experiments involving IAV and *S. pneumoniae* were performed within the Biosafety Level 2 facility of the Institut Pasteur de Lille. The protocols were validated by the local committee for the evaluation of the biological risks and complied with current national and institutional regulations and ethical guidelines (Institut Pasteur de Lille/B59- 350009). The experimental protocols using animals were approved by the institutional ethical committee, Comite d’Ethique en Experimentation Animale (CEEA) 75, Nord Pas-de-Calais. The animal study was authorized by the Education, Research and Innovation Ministry under registration number APAFIS#13743-2018022211144403v2 and APAFIS#33193-2021092318476492.

### F. duncaniae culture and treatment

*F. duncaniae* A2–165 (DSM 17677, DSMZ collection, Braunschweig, Germany) and I-4574 (CNCM collection, Paris, France) strains, both isolated from human fecal stool, were grown at 37°C in Brain-heart infusion medium supplemented with 0.5% yeast extract (Difco, Detroit, USA), 2 mM acetate, 2 mg/ml fructose and 0.5 mg/ml cysteine HCl (Sigma-Aldrich), in an anaerobic chamber (90% N2, 5% CO,2 and 5% H2). Bacteria were washed and pellets were resuspended in PBS containing 16% glycerol at 5 x 10^9^ CFU/ml aliquoted and frozen at -80°C until use. Mice were daily treated by intragastric administration (200 µl) of PBS containing 16% glycerol (mock-infected and IAV-infected mice) or of a suspension of *F. duncaniae* at 5 x 10^9^ CFU/ml (1 x 10^9^ CFU/mouse/day), 5 days before infection and until the end of the procedure. Pasteurization was obtained after 30 min at 70°C. Integrity of the bacteria was checked by microscopy analysis.

### Body weight measurement and tissue collection

Body weight was monitored daily after IAV infection. Mice were sacrificed at 2 dpi or 7 dpi. Bronchoalveolar lavage fluids were collected using 0.5 ml PBS containing 0.2% BSA and centrifuged at 1100 x g for 2 min at 4°C. Supernatants were stored at -20°C until ELISA or Multiplex analysis. After perfusion with PBS, the left lobe of the lung was stored in 4% PBS-buffered formaldehyde for 2 days, rinsed in PBS, transferred into 70% ethanol solution and processed into paraffin-embedded tissue blocks for histology assessment. Part of the right lobe was stored in RNAlater^®^ (Ambion, Thermo Fisher Scientific, Waltham, MA) and frozen at -80°C until qRT-PCR analysis. The remaining part of the lung was immediately frozen in liquid nitrogen and stored at -80°C until TCID_50_ evaluation. Colon length was measured as described in (29). Fecal samples were collected from each individual mouse, immediately frozen in liquid nitrogen and stored at -80°C until SCFA quantification and 16S rRNA gene amplicon sequencing.

### Quantification of viral loads in lungs

Virus load in lung homogenates was assessed by titration of 50% tissue culture infectious dose (TCID50) using Madin-Darby canine kidney (MDCK) cells. Quantification of viral load and of gene expression was as follows. After homogenization of lung and colon tissue in RA1 buffer (Macherey-Nagel, Hoerdt, Germany), total RNA was purified using the NucleoSpin RNA isolation kit (Macherey-Nagel). The quantity and quality of RNA were checked using a NanoDrop spectrophotometer (Thermo Fisher Scientific). RNA was reverse transcribed using the High-Capacity RNA-to-cDNA™ Kit (Applied Biosystems, Hammonton, NJ). Quantification of the viral load was determined by measuring the copy number of RNA encoding the viral *M1* gene. Briefly, after treatment with RNAse OUT (Invitrogen), RNA was reverse-transcribed using the SuperScript^®^ II Reverse Transcriptase (Invitrogen, Life Technologies, Carlsbad) and primer specific for *M1* (5′-TCT AAC CGA GGT CGA AAC GTA-3′). Amplification was performed using TaqMan Universal PCR Master Mix (Applied Biosystems), the specific primers for *M1* (forward: 5′-AAG ACC AAT CCT GTC ACC TCT GA-3′; reverse: 5′-CAA AGC GTC TAC GCT GCA GTC C-3′) and *M1* specific TaqMan probe (FAM) 5′-TTT GTG TTC ACG CTC ACC GTG CC-3′ (TAMRA), on the QuantStudio™ 5 Flex Real-Time PCR System (Applied Biosystems). The standard curve was performed using a plasmid constructed with a synthetic gene containing the IAV M1 sequence (segment 7).

### Assessment of gene expression by quantitative RT-PCR

After homogenization and RNA extraction of lung and intestinal (colon) tissues, RNA was reverse transcribed using the High-Capacity RNA-to-cDNA™ Kit (Applied Biosystems). qRT-PCR was performed using the Power SYBR Green PCR Master Mix on the QuantStudio™ 5 Flex Real-Time PCR System (Applied Biosystems) (**Table 1** for the sequences of the oligonucleotides). The relative gene expression (2^-▵▵^CT) was normalized according to the PCR cycle thresholds (Ct) for the gene of interest and the housekeeping gene coding glyceraldehyde 3-phosphate dehydrogenase (Gapdh) (^▵^Ct) and to the ^▵^Ct values between non-infected (mock) and infected animals.

**Table 1:** Sequences of oligonucleotides used in the present study.

Oligonucleotides	
*Mx1*	Forward 5'-AGAAGGTGCGGCCCTGTATT-3'
	Reverse 5'-TGAACTCTGGTCCCCAATGACA-3'
*Isg15*	Forward 5'-GGCCACAGCAACATCTATGAGG-3'
	Reverse 5'-CTCGAAGCTCAGCCAGAACTG-3'
*Ifnb*	Forward 5'-TGGGTGGAATGAGACTATTGTTG-3'
	Reverse 5'-CTCCCACGTCAATCTTTCCTC-3'
*Ifng*	Forward 5'-CAACAGCAAGGCGAAAAAG-3'
	Reverse 5'-GTGGACCACTCGGATGAGCT-3'
*Tnfa*	Forward 5'-CATCTTCTCAAAATTCGAGTGACAA-3'
	Reverse 5'-TGGGAGTAGACAAGGTACAACCC-3'
*Il1b*	Forward 5'-TCGTGCTGTCGGACCCATA-3'
	Reverse 5'-GTCGTTGCTTGGTTCTCCTTGT-3'
*Il6*	Forward 5'-CAACCACGGCCTTCCCTACT-3'
	Reverse 5'-CCACGATTTCCCAGAGAACATG-3'
*Ccl2*	Forward 5'-GCAGCAGGTGTCCCAAAGAA-3'
	Reverse 5'-TCATTTGGTTCCGATCCAGGT-3'
*Cxcl2*	Forward 5'-GAAGTCATAGGCACTCTCA-3'
	Reverse 5'-TTCCGTTGAGGGACAGCA-3'
*FoxJ1*	Forward 5'-CCACCAAGATCACTCTGTCGG-3'
	Reverse 5'-AGGACAGGTTGTGGCGGAT-3'
*Cc10*	Forward 5'-CATCTGCCCAGGATTTCTTCAA-3'
	Reverse 5'-CGCATTTTGCAGGTCTGAGC-3'
*Zo1*	Forward 5'-AGGTCTTCGCAGCTCCAAGAGAAA-3'
	Reverse 5'-ATCTGGCTCCTCTCTTGCCAACTT-3'

### Bronchoalveolar lavage fluid collection and determination of cytokine and chemokine levels

Bronchoalveolar lavage was performed by instillation of 500 μL of PBS containing 2% bovine serum albumin via the exposed trachea into the lungs, followed by aspiration after 30 s, using a 18G IV cannula (B. Braun, Melsungen, Germany). Supernantants were centrifuged at 10,000 g for 2 minutes before cytokine dosage. The concentrations of IFN Λ2−3 was determined by ELISA (R&D systems DuoSet, Minneapolis, MN). Concentrations of IFN-γ, IFN-β, IFN-α, TNF-α, and CCL2 were determined using multiplex analysis following the manufacturers’ instructions (LEGENDplex, Biolegend, San Diego, CA).

### Lung histology

Lung tissue samples were fixed in 10% PBS-buffered formaldehyde for 48h, rinsed in PBS, transferred in 70% ethanol and then paraffin-embedded under standardized conditions. Tissue sections (3 µm thick) were stained with hematoxylin and eosin. Lung sections were scanned with a Nanozoomer (Hamamatsu Photonics, city, Hamamatsu, Japan), and morphological changes were assessed using a semi-quantitative dual histopathology score adapted from (93, 94), which evaluates each following score: cellular death/necrosis, alveolar and/or perivascular edema, hyaline membrane or fibrin, inflammation, thrombi, congestion, hemorrhage, type II hyperplasia, and syncytia from 0 to 4 (0 = absent, 1 = 1-10% of the lung section affected, 2 = 11-25% of the lung section affected, 3 = 26-50% of the lung section affected, and 4=>50% of the lung section affected). The degree of peribronchial and perivascular inflammation was scored on a subjective scale of 0 (no) to 5 (severe) in a blind manner. The percentage of the altered/inflamed zone of the section (lung lesion) was also evaluated (from score 0 to score 10). The score was then defined as the sum of the peribronchial and perivascular scores, and the percentage of altered zone. Representative photomicrographs were taken to illustrate the major distinguishing morphological features among the experimental groups.

### Sample collection, genomic DNA extraction and sequencing

Genomic DNA extraction from feces was conducted following an adaptation from González-Cabrera et al. (95). Briefly, around 50 mg of feces were crushed in 300 µl of SLX-Mlus Buffer (Omega Bio-tek, Norcross, GA) using a plastic homogenizer. A volume of 50 µl was then sampled and incubated at 99°C for 10 min with 16.7 µl of 1 M NaOH. The solution was neutralized adding 66.7 µL of 0.1248 M HCl, 0.125 M Tris-HCl and 0.5% Triton X-100 before a last incubation at 99°C for 10 min. Genomic DNA suspensions were stored at -20°C. Microbial diversity and composition were determined for each sample by targeting a portion of the ribosomal genes. A set of 12 primers (forward) and 3 primers (reverse) were used to amplify the V3 and V4 hypervariable region of the 16S rRNA gene fragment using an optimized 16S-amplicon-library preparation protocol (Biomnigene, Besançon France). Briefly, 16S rRNA gene PCR was performed using 5 µl of 1/40 diluted genomic DNA using GoTaq^®^ Rapid PCR Master Mix (Promega, Madison, WI) using 15 barcoded primers at final concentrations of 0.2 μM and an annealing temperature of 55°C for 38 cycles. The PCR products were multiplexed at equal concentrations and purified using a PippinHT system (Sage Science, Beverly, MA). Sequencing was performed using a 250-bp paired-end sequencing protocol on an Illumina MiSeq platform (Illumina, San Diego, CA).

### Gut microbiota analysis

A step of removal of low-quality reads from the raw paired-end reads were performed using Fastp. Remaining sequences were assigned to samples based on barcode matches using cutadapt (version 4.4). Data were then imported in Qiime2 (2023.7) and forward and reverse primer sequences were removed using the cutadapt plugin. The sequences were denoized using the DADA2 method, and reads were classified using the GTDB database (release 214). A total of 666,810 paired-end reads were analyzed, with an average of 22,227 per sample (range: 10,492 to 47,244). Beta diversity was computed using Qiime2. Principal Coordinate analyses of the Weighted Unifrac distance were performed to assess beta diversity. Differences between groups were tested using PERMANOVA analysis. Raw sequence data are accessible in the National Center for Biotechnology Information (project number PRJNA1046012), biosample accession numbers SAMN38468615 to SAMN38468644 (https://dataview.ncbi.nlm.nih.gov/object/PRJNA1046012?reviewer=7da92vicemsbkcq6oqsf79pbph). Differential analysis was performed using the linear discriminant analysis effect size (LEfSe) pipeline.

### Measurement of SCFA concentrations in the cecal contents

Fecal concentrations of SCFAs were quantified as previously described (28). After extraction of fecal contents with water (wt g/vol) and centrifugation at 12,000 × g for 15 min, the proteins were precipitated from the suspensions using a phosphotungstic acid saturated solution. Supernatants (100 µl) were analyzed using a gas chromatograph (GC7890B; Agilent Technologie, Les Ulis, France). All samples were analyzed in duplicate. Data were collected and peaks integrated using the Open Lab Chemstation software (Agilent Technology, Santa Clara, CA). Results were expressed as μmol/gramm of fecal content.

### Statistical analyses

For infectious markers, results are expressed as mean ± standard error of the mean (SEM). Statistical analyses were performed using GraphPad Prism v8.0.2 and R v4.0.2 softwares. The Mann-Whitney *U* test was used to compare the two groups, unless otherwise stated. Comparisons of more than two groups were analyzed using the one-way ANOVA Kruskal-Wallis test (nonparametric), followed by Dunn’s post-test. *P < 0.05; ** *P* < 0.01, *** *P* < 0.001.

## Data availability statement

The datasets presented in this study can be found in online repositories. The names of the repository/repositories and accession number(s) can be found in the article/**Supplementary Material**.

## Ethics statement

The animal study was approved by Comite d’Ethique en Experimentation Animale (CEEA) 75, Nord Pas-de-Calais. The study was conducted in accordance with the local legislation and institutional requirements.

## Author contributions

LC: Investigation, Methodology, Writing – review & editing. SH: Investigation, Methodology, Writing – review & editing. LuD: Investigation, Writing – review & editing. FB: Investigation, Methodology, Writing – review & editing. LoD: Investigation, Writing – review & editing. VR: Investigation, Methodology, Writing – review & editing. M-HG: Investigation, Writing – review & editing. MP: Investigation, Writing – review & editing. VS: Investigation, Writing – review & editing. CR: Investigation, Writing – review & editing. IW: Conceptualization, Investigation, Writing – review & editing. HS: Conceptualization, Writing – review & editing. SA: Investigation, Writing – review & editing. AD: Investigation, Writing – review & editing. PL: Conceptualization, Writing – review & editing. J-MC: Conceptualization, Investigation, Writing – review & editing. CG: Conceptualization, Investigation, Writing – original draft. FT: Conceptualization, Funding acquisition, Investigation, Supervision, Writing – original draft.

## Supplementary material

Supplementary Figure 1 Impact of I-4574 supplementation in IAV-infected mice on the composition of the gut microbiota and on the production of SCFAs. **(A)** Seven days after IAV infection, fecal contents were collected for 16S rRNA profiling. Fecal samples from each mock-infected mouse were also collected. The relative frequency of some bacterial phyla is depicted. **(B)** Taxonomic (class) composition of the fecal microbiota. **(A, B)** (n = 10/group, one of two independent experiments shown). **(C)** Cecal contents were collected for SCFA quantification. Results are expressed as mean ± SEM (n = 5, mock and n = 9-11, IAV) **(D)**. **(D)** Cecal contents were collected for SCFA quantification. Results are expressed as mean ± SEM (n = 5, mock and n = 9-11, IAV). **(A, C)**, Significant differences were determined using the One-way ANOVA Kruskal-Wallis test (nonparametric), followed by the Dunn’s posttest test (* *P* < 0.05, ** *P* < 0.01, *** *P* < 0.001).
