# Supplementary figures and images for: Faecalibacterium duncaniae as a novel next generation probiotic against influenza
Source: Front Immunol. 2024 Mar 12;15:1347676. doi: 10.3389/fimmu.2024.1347676 (PMC11000806; doi:10.3389/fimmu.2024.1347676)

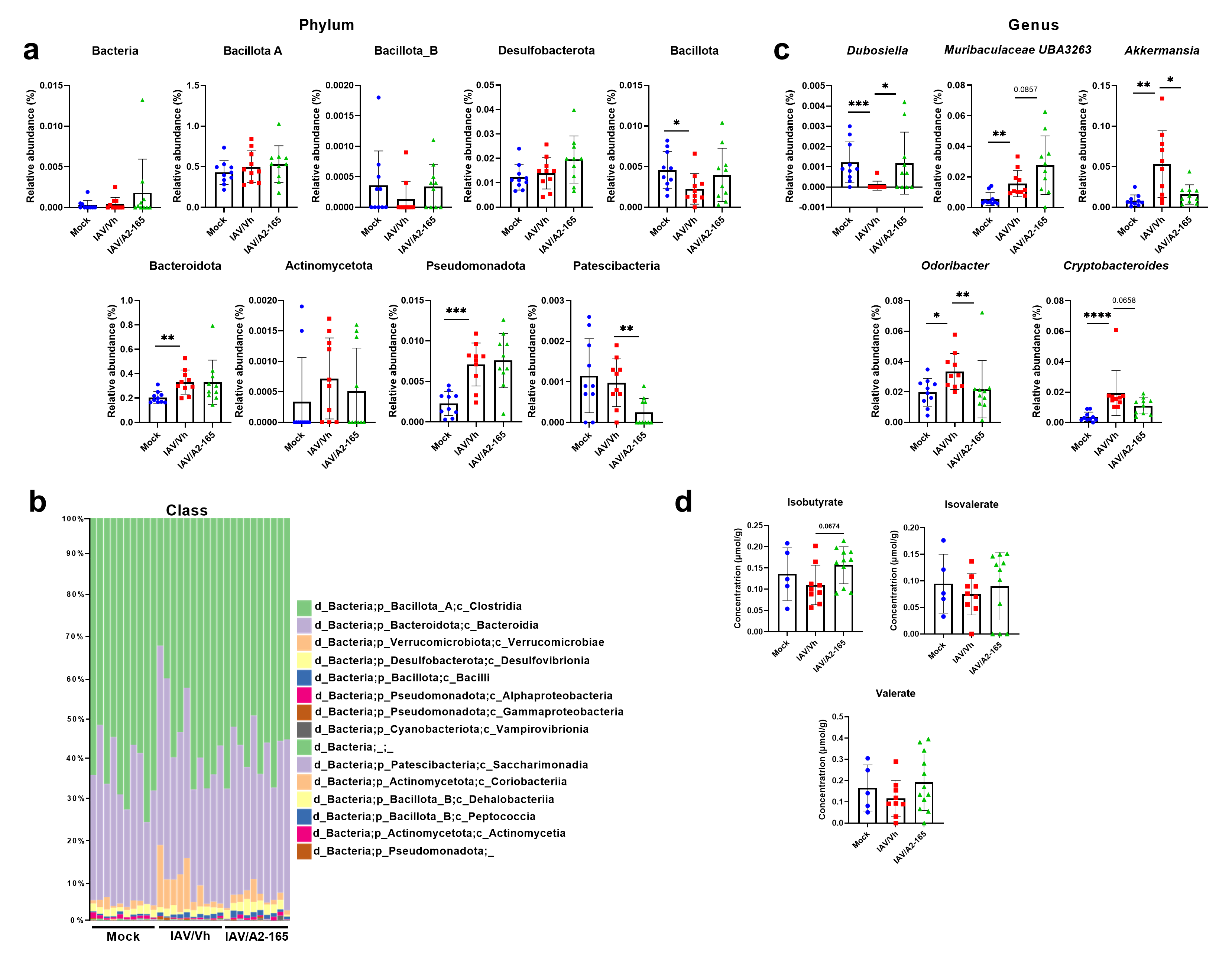

Supplement: Supplementary Figure 1 — Impact of I-4574 supplementation in IAV-infected mice on the composition of the gut microbiota and on the production of SCFAs. (A) Seven days after IAV infection, fecal contents were collected for 16S rRNA profiling. Fecal samples from each mock-infected mouse were also collected. The relative frequency of some bacterial phyla is depicted. (B) Taxonomic (class) composition of the fecal microbiota. (A, B) (n = 10/group, one of two independent experiments shown). (C) Cecal contents were collected for SCFA quantification. Results are expressed as mean ± SEM (n = 5, mock and n = 9-11, IAV) (D). (D) Cecal contents were collected for SCFA quantification. Results are expressed as mean ± SEM (n = 5, mock and n = 9-11, IAV). (A, C), Significant differences were determined using the One-way ANOVA Kruskal-Wallis test (nonparametric), followed by the Dunn’s posttest test (* P < 0.05, ** P < 0.01, *** P < 0.001). [file Image_1.tif]
